# Whip-LAMP: a novel LAMP assay for the detection of *Trichuris muris*-derived DNA in stool and urine samples in a murine experimental infection model

**DOI:** 10.1186/s13071-020-04435-1

**Published:** 2020-11-07

**Authors:** Pedro Fernández-Soto, Carlos Fernández-Medina, Susana Cruz-Fernández, Beatriz Crego-Vicente, Begoña Febrer-Sendra, Juan García-Bernalt Diego, Óscar Gorgojo-Galindo, Julio López-Abán, Belén Vicente Santiago, Antonio Muro Álvarez

**Affiliations:** Infectious and Tropical Diseases Research Group (e-INTRO), Biomedical Research Institute of Salamanca-Research Centre for Tropical Diseases at the University of Salamanca (IBSAL-CIETUS), Faculty of Pharmacy, University of Salamanca, Salamanca, Spain

**Keywords:** *Trichuris trichiura*, LAMP, *Trichuris muris*, Human trichuriasis, Urine

## Abstract

**Background:**

*Trichuris trichiura* (human whipworm) infects an estimated 477 million individuals worldwide. In addition to *T. trichiura*, other *Trichuris* species can cause an uncommon zoonosis and a number of human cases have been reported. The diagnosis of trichuriasis has relied traditionally on microscopy. Recently, there is an effort to use molecular diagnostic methods, mainly qPCR. LAMP technology could be an alternative for qPCR especially in low-income endemic areas. *Trichuris muris*, the causative agent of trichuriasis in mice, is of great importance as a model for human trichuriasis. Here, we evaluate the diagnostic utility of a new LAMP assay in an active experimental mouse trichuriasis in parallel with parasitological method by using stool and, for the first time, urine samples.

**Methods:**

Stool and urine samples were collected from mice infected with eggs of *T. muris*. The dynamics of infection was determined by counting the number of eggs per gram of faeces. A LAMP based on the *18S* rRNA gene from *T. muris* was designed. Sensitivity and specificity of LAMP was tested and compared with PCR. Stool and urine samples were analysed by both LAMP and PCR techniques.

**Results:**

*Trichuris muris* eggs were detected for the first time in faeces 35 days post-infection. LAMP resulted specific and no cross-reactions were found when using 18 DNA samples from different parasites. The detection limit of the LAMP assay was 2 pg of *T. muris* DNA. When testing stool samples by LAMP we obtained positive results on day 35 p.i. and urine samples showed amplification results on day 20 p.i., i.e. 15 days before the onset of *T. muris* eggs in faeces.

**Conclusions:**

To the best of our knowledge, we report, for the first time, a novel LAMP assay (Whip-LAMP) for sensitive detection of *T. muris* DNA in both stool and urine samples in a well-established mice experimental infection model. Considering the advantages of urine in molecular diagnosis in comparison to stool samples, should make us consider the possibility of starting the use urine specimens in molecular diagnosis and for field-based studies of human trichuriasis where possible. Further studies with clinical samples are still needed.
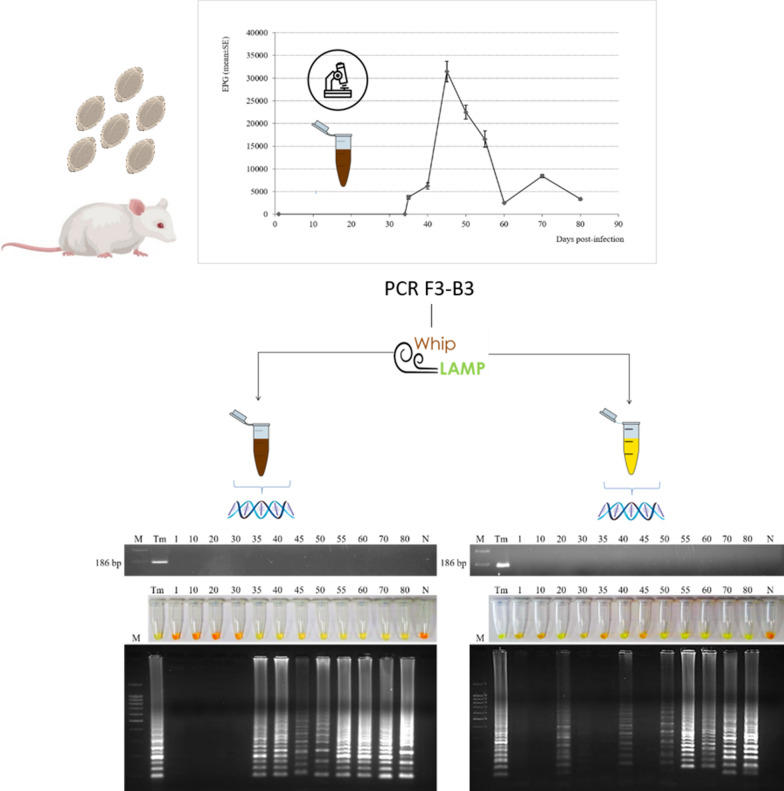

## Background

Soil-transmitted helminth (STH) infections refer to diseases mainly caused by intestinal nematodes, *Ascaris lumbricoides* (roundworm), *Ancylostoma duodenale* and related species, and *Necator americanus* (hookworms), and *Trichuris trichiura* (whipworm) affecting almost 2 billion people worldwide, commonly in tropical and subtropical countries among populations living in poverty and inadequate sanitation [1]. Another important STH is *Strongyloides stercoralis* (threadworm), but it is usually omitted in clinical practices and control programmes because it needs different diagnostic tools and different treatment [2]. The World Health Organization (WHO) estimates STH infections contribute 5.18 million disability-adjusted life-years (DALYs) worldwide in 2010 [3] with at least 12,000 deaths each year [4], and identifies three population groups at high risk: school-aged children; pre-school children; and girls and women of reproductive age [5].

Within this group of STHs, *T. trichiura* is considered the third most common zoonotic nematode to infect humans with an estimated 477 million people infected worldwide, mostly school-aged children, with 13% contribution to DALYs for intestinal helminths [6, 7]. Heavy whipworms burdens cause morbidity through dysentery (*Trichuris* dysentery syndrome), with symptoms including stomach pain, diarrhoea and in extreme cases, rectal prolapse. Infected children usually show signs of malnutrition, stunted growth, intellectual retardation and educational deficits. Additionally, infection during pregnancy increases the risk of maternal anaemia and reduces infant birth weight and survival [1].

In addition to *T. trichiura*, other *Trichuris* species, namely, *T. suis* and *T. vulpis* (causing trichuriasis in pigs and canids, respectively) can cause an uncommon zoonosis and a number of human cases have been reported [8]. The use of *T. suis* ova administration as therapy for inflammatory bowel disease [9, 10], has recently stimulated investigations into systematics of *Trichuris* species from human and non-human primates, and other related species and their zoonotic potential. A complex scenario with multiple *Trichuris* species that may be shared between humans and non-human primates with implications in the control of whipworms infections has been suggested recently [11].

On the other hand, the causative agent of trichuriasis in mice, the closely related *T. muris* parasite, is of great importance in research as a well-established experimental model in the laboratory for the development of new treatments and diagnostic tools for human trichuriasis and to study the host’s immune response [12, 13]. In both mice and human hosts, ingested whipworm embryonated eggs from the external environment hatch into larvae in the large intestine and establish infection within the epithelium of the cecum and colon facilitated by the interaction with the intestinal microflora [14]. Typically, following four molts, dioecious adult whipworms develop to patency, mate and intermittently release unembryonated eggs into the environment with the stool.

In uncomplicated cases of human trichuriasis, the copro-microscopic detection for identifying the characteristic morphology of the lemon-shaped *T. trichiura* eggs using the Kato-Katz technique is usually sufficient with some limitations as for ascariasis [15]. However, the Kato-Katz thick smear-based stool examination is highly observer-dependent and lacks sensitivity, especially in low-intensity infections and specifically when approaching the end of the elimination phase in a control setting [16, 17]. Other direct diagnostic methods to detect *T. trichiura* eggs are used, such as the McMasterʼs method, flotation techniques (FLOTAC and Mini-FLOTAC) and bio-molecular methods [16]. A drawback of these methods is that most require fresh stool samples, which is a limitation in large epidemiological studies. The diagnostic performance of Kato-Katz using stool samples fixed with sodium acetate (SAF) was recently suggested as a good option for transporting samples for diagnosis, especially in rural areas in developing countries [18]. As for other STH infections, serology could have a role in diagnosis of trichuriasis in some situations where stool samples are unavailable using either antigen or antibody-detection assays [19]. Nevertheless, serology might overestimate the prevalence of the infection due to cross-reactivity with other nematode infections. To solve these problems, there has been an increasing effort in research to use molecular diagnostic assays for STH (mainly PCR, nested-PCR and qPCR) [20], although the different methods have been basically developed for epidemiologic studies and for the diagnosis of returning travellers in high-income countries [21, 22]. Moreover, many of the PCR and qPCR techniques are multiplexed or multi-parallel thus increasing the ability to detect multiple concurrent infections when compared with microscopy [20]. Specifically, for molecular detection of *T. trichiura*, real-time qPCR methods have been tested targeting different gene markers, including sequences of the internal transcribed spacers 1 (ITS1) [23] and 2 (ITS2) [24] and sequence repeats [25]; these have shown results with higher sensitivity than detection by microscopy [26]. However, the real-time qPCR-based methods currently have limitations that make them not applicable in field contexts and settings such as the need for trained scientific personnel [27]. Regarding the specificity of the detection, it has been suggested that when highly-specific sequences to detect *T. trichiura* are targeted by qPCR, other closely related *Trichuris* species suspected of being potentially zoonotic (e.g. *Trichuris ovis*) may be missed [25].

Loop-mediated isothermal amplification (LAMP) assay based on detection of nucleic acids is easy to perform, relatively inexpensive, and has been successfully used for detection of many pathogenic agents [28, 29], including a large number of helminths that cause human diseases [30]. Nevertheless, very few conventional LAMP assays for the detection of STH have been developed to date, including *Necator americanus* [31], *Ascaris lumbricoides* [32], and *Strongyloides stercoralis* [33, 34]. For *T. trichiura*, a particular type of LAMP assay with unique asymmetrical primer design that makes the assay highly specific under isothermal conditions, called smart amplification process (SmartAmp2), has been also used for the specific detection of *N. americanus* and *A. lumbricoides* [35]. Most recently, a specific LAMP to detect exclusively *T. trichiura* in faecal samples has been developed with a demonstrated 88% specificity and 77% sensitivity when compared with Kato-Katz using faecal samples collected from school children in Kenya [36]. All these molecular assays based on LAMP technology have been developed and optimized to detect DNA of the parasites in faecal extracts, with the only exception to date of *Strongyloides* spp., which has also been detected in urine samples both taken from an experimental rodent model of strongyloidiasis [34] and patients [37] in previous studies of our research group.

In this study, we examined the diagnostic utility of a new designed LAMP assay in an active experimental rodent trichuriasis in parallel with a parasitological detection method (direct faecal examination). We used as a source for *Trichuris* spp. DNA detection both stool and, to the best of our knowledge, for the first time, urine samples from mice experimentally infected with *T. muris* eggs. The LAMP assay developed here (named Whip-LAMP) was shown to be sensitive and specific in detecting *Trichuris* spp. DNA.

## Methods

### Animals and experimental infection

#### Mice

Six 7-week-old female CD1 mice (Charles River Laboratories, Lyon, France) weighing 22–24 g where used as the source for urine and stool samples throughout the experiments. Animals were divided into two groups of 6 mice each and then housed in pathogen-free animal experimentation facilities of the University of Salamanca (Salamanca, Spain) in standard ventilated polycarbonate, and placed in a humidity (30% to 70%) and temperature (22–25 °C) controlled environment with a 12 h light-dark cycle and had free access to food and water.

#### Trichuris muris experimental infection

*Trichuris muris* (Edinburg strain) eggs to infect mice were kindly provided by the personnel of the Department of Parasitology of the Complutense University of Madrid, Madrid, Spain. The infective eggs were kept in sterile water until use. Three mice were infected with a high (*n* = 300) dose of eggs each in sterile-filtered (0.2 µm) water by oral gavage. Infected mice were treated with dexamethasone (Fortecortin, Merk, SL., Madrid, Spain) in drinking water (0.3 mg/l) after Gómez-Samblas et al. [38]. Six mice were not infected and served as healthy controls. During the 80-day infection, the two groups of mice were housed in separate cages. After the experiments, infected mice were humanely euthanized by CO_2_ inhalation in a gas chamber followed by terminal exsanguination.

### Mice samples, processing and monitoring *Trichuris muris* infection

Stool and urine samples from infected mice were collected on days 1, 10, 20, 30, 35, 40, 45, 50, 55, 60, 70 and 80 post-infection (p.i.). Stool and urine samples from uninfected mice were collected when required. To collect samples, each mouse was removed from the cage and housed individually in a plastic beaker cleaned with 70% v/v ethanol. To avoid cross-specimen contamination (urine in faeces or faeces in urine), after urine samples collection, each mouse was removed to another clean plastic beaker cleaned with 70% v/v ethanol for the collection of faeces. Each animal was returned to its home cage as soon as samples were collected.

#### Mice urine samples

Urine samples (about 40–100 µl per infected mouse) were collected at days mentioned above using a pipette either from a plastic beaker or by gently stroking the lower side of the abdomen and transferred to a 1.5 ml tube. Later, the 6 urine samples obtained were pooled in a mix and stored immediately at -20 °C for further DNA extraction.

#### Mice stool samples

Approximately, 3–4 faecal pellets per infected mouse were also collected with tweezers from the plastic beaker and transferred into 1.5 ml tubes at days mentioned above. The 6 stool samples where then pooled, and the resulting mix was treated as a single sample that was subsequently divided into two fractions: one was freeze-dried stored at -20 °C for further DNA extraction; and another was weighed and kept at room temperature by adding 1 ml of 3% formalin to preserve parasite eggs for later counting in a McMaster chamber. Stool and urine samples from uninfected mice were also collected and pooled when required, following the same procedure described for infected mice.

The dynamics of *T. muris* infection was determined by counting the number of eggs per gram of faeces (EPG) in a McMaster chamber on days 1, 10, 20, 30, 35, 40, 45, 50, 55, 60, 70 and 80 p.i. Three readings were made of each pooled mix of faeces from infected animals preserved in formalin to obtain the average. The results were expressed as mean ± SE.

### DNA extraction for molecular analysis

#### Parasite DNA samples

*Trichuris muris* adult worms preserved in ethanol were used as source for DNA extraction. Genomic DNA (gDNA) was extracted using the NucleoSpin Tissue Kit (Macherey-Nagel, GmH & Co., Düren, Germany) following the manufacturers’ instructions. DNA obtained was measured by using a Nanodrop ND-1000 spectrophotometer (Nanodrop Technologies, Wilmington, USA), and then diluted with ultrapure water to a final concentration of 10 ng/μl. Serial 10-fold dilutions were prepared with ultrapure water ranging from 1 × 10^−1^ to 1 × 10^−10^ and stored at -20 °C until use. The gDNA thus prepared was used as positive control in all PCR and LAMP reactions and for assessing sensitivity of both molecular assays.

To determine the specificity of molecular methods to amplify only *T. muris* DNA, a total of 18 DNA samples available in our laboratory from several helminths and protozoans were used as miscellaneous control samples, including *Schistosoma mansoni*, *S. haematobium*, *Fasciola hepatica*, *Amphimerus* sp., *Echinococcus granulosus*, *Taenia saginata*, *T. solium*, *Dicrocoelium dendriticum*, *Hymenolepis diminuta*, *Anisakis simplex*, *Brugia pahangi*, *Loa loa*, *Mansonella perstans*, *Strongyloides venezuelensis*, *Ascaris suum*, *Giardia duodenalis*, *Cryptosporidium parvum* and *Entamoeba histolytica.* These DNA samples (0.5 ng/μl) were stored at -20 °C until use in amplification trials.

#### DNA from mice urine samples

A volume of 200 µl of pooled urine samples from infected mice corresponding to days of collection and a pool of urine samples from non-infected mice were used to extract the DNA using the i-genomic Urine DNA Extraction Mini Kit (Intron Biotechnology, Sungnam-si, Korea) following the manufacturers’ instructions. Once extracted, the purified DNA was stored at -20 °C until analysis.

#### DNA from mice stool samples

The pooled frozen faecal pellets from infected mice (200 mg/each) corresponding to days of collection and also a pool of faecal pellets from non-infected mice (200 mg) were used for DNA extraction using the NucleoSpin Tissue Kit (Machery-Nagel, GmbH & Co) according to the modified protocol recommended for stool samples - following the manufacturers’ instructions. All DNA samples obtained were stored at -20 °C until use in molecular assays.

### Design of primers for the LAMP assay

Based on the bioinformatics analysis, two sequences of DNA corresponding to a linear genomic DNA partial sequence in the nuclear small subunit rRNA gene (*18S* rDNA) from *T. muris* were selected and retrieved from GenBank to be used for the design of primers: a sequence of 1845 bp (GenBanK: AF036637.1) [39], and another of 1829 bp (GenBank: HF586907.1) [40]. An alignment for the two selected sequences using ClustalW [41] resulted in a consensus sequence of 1847 bp for *T. muris.*

On the other hand, a total of 5 sequences corresponding to a linear genomic DNA partial sequence in the *18S* rRNA gene fitting to 5 *T. trichiura* isolates were also selected and retrieved from GenBank, including isolate TST332 (GQ352555.1; 2582 bp), isolate TST214 (GQ352554.1; 2561 bp), isolate TH1 (GQ352553.1; 2576 bp), isolate D42 (GQ352552.1; 2585 bp), and isolate TH2 clone E (GQ352551.1; 2567 bp). In this case, a multiple sequence alignment using ClustalW generated a consensus sequence of 1846 bp for *T. trichiura.*

Next, the two consensus sequences obtained for *T. muris* and *T. trichiura,* respectively, were compared and a 1910 bp final common consensus sequence was obtained. Subsequently, a BLASTN search and alignment analysis [42] indicated that the 1910 bp resulting final consensus sequence had 93% similarity with the sequence reported for the *18S* rRNA gene for *T. muris* (GenBank: HF586907.1) and a 94% similarity with a partial sequence for the *18S* rRNA gene for *T. trichiura* isolate TH2 clone B (GenBank: GQ352548.1). No regions of similarity between this consensus sequence and other sequences reported for possible human pathogens were detected. The presence of the 1910 bp sequence selected was finally confirmed *in silico* in the currently available genome annotated in WormBase Parasite [43] for *T. muris* (Bioproject PRJEB126) and for *T. trichiura* (Bioproject PRJEB535).

LAMP primers to amplify DNA of *T. muris* and *T. trichiura* were designed based on the 1910 bp consensus sequence using the Primer Explorer software program [44] Several potential LAMP primer sets were generated and further refinement in design was manually developed following the instructions described in “A guide to LAMP primer designing” [45]. The set of primers finally selected is indicated in Table 1.

**Table 1:** Sequences of LAMP primers for the amplification of a partial consensus sequence based on the linear genomic DNA partial sequence in the *18S* rDNA from *Trichuris muris* and *T. trichiura*

Primer	Length (bp)	Sequence (5′–3′)
F3	20	ACTTTCGATGGTACGCTACG
B3	20	GCGTCTCATGGAGAATCGTT
FIP (F1c+F2)	40	TCTCAGGCTCCCTCTCCGGA-TGCTTACCATGGTGACAACG
BIP (B1c+B2)	41	GGCTACCACATCCAAGGAAGGC-GTCACTACCTCCCCGATCT

*Abbreviations*: F3, forward outer primer; B3, reverse outer primer; FIP, forward inner primer (comprising F1c and F2 sequences); BIP, reverse inner primer (comprising B1c and B2 sequences)

### PCR using outer primers F3 and B3

The outer LAMP primers F3 and B3 (Table 1), were initially tested for the detection of *T. muris* DNA by a touchdown-PCR (TD-PCR) protocol to verify whether the *in silico* expected target was amplified. All PCR assays were carried out in 25 μl reaction mixture containing 2.5 μl of 10× buffer, 1.5 μl of 25 mmol/l MgCl_2_, 2.5 μl of 2.5 mmol/l dNTPs, 0.5 μl of 100 pmol/l F3 and B3, 2 U *Taq* polymerase and 2 μl of DNA template. Initial denaturation was conducted at 94 °C for 1 min, followed by a touchdown program for 15 cycles with successive annealing temperature decrements of 1 °C every 2 cycles. For these 2 cycles, the reaction was denatured at 94 °C for 20 s, followed by annealing at 64 °C–58 °C for 20 s and polymerization at 7 °C for 30 s. The following 15 cycles of amplification were similar, except that the annealing temperature was 57 °C, with a final extension at 72 °C for 10 min.

The specificity of PCR was tested with heterogeneous DNA samples from other parasites included in the study. The sensitivity of the TD-PCR (F3-B3) to establish the detection limit of *T. muris* DNA was assayed with 10-fold serial dilutions prepared as previously mentioned. Negative controls (ultrapure water, urine or stool from uninfected mice) and positive controls (genomic DNA from *T. muris*) were included in all trials. The PCR products were subjected to 1.5–2% agarose gel electrophoresis stained with a safe DNA Gel Stain (Genetics, GmbH, Sachsen-Anhalt, Germany) and visualized under UV light.

### LAMP assay: analytical specificity, limit of detection and evaluation

LAMP reactions were performed using the selected primers in a total volume of 25 μl. Reaction mixtures consisted of 40 pmol each of FIP and BIP primers, 5 pmol each of F3 and B3 primers, 1.4 mM each of dNTP (Intron Biotechnology, Sungnam-si, Korea), 1× isothermal amplification buffer: 20 mM Tris-HCl (pH 8.8), 50 mM KCl, 10 mM (NH4)2SO_4_, 2 mM MgSO_4_, 0.1% Tween20 (New England Biolabs, Hitchin, UK) and betaine (0.8, 1, 1.2, 1.4 or 1.6 M) (Sigma-Aldrich, St Louis, USA), supplementary MgSO_4_ (2, 4, 6 or 8 mM) (New England Biolabs), 8 U of *Bst* polymerase 2.0 WarmStart (New England Biolabs), 2 μl of template DNA, and nuclease-free water was added up to a final volume of 25 μl. To establish a standard protocol for the LAMP assay, the reaction was carried out by incubating the tubes in a heating block at different temperatures (61, 63 or 65 °C) for 45–60 min and then heated at 80 °C for 5–10 min to terminate the reaction.

To determine the specificity of the LAMP assay, DNA samples from other parasites included in the study were analysed. To assess the lower detection limit of the LAMP assay, a series of decimal dilutions of genomic DNA from *T. muris* as mentioned above were tested in comparison with amplification by PCR (F3-B3).

Visual detection of the LAMP results was performed by adding 2 µl of 1:10 diluted 10,000× concentration SYBR Green I (Invitrogen, Carlsbad, California, USA) to the reaction tubes post-amplification. Colour changes in LAMP reactions were observed in natural light, with the naked eye. Green fluorescence was observed in a successful reaction, whereas it remained its original orange colour in the negative reaction. The LAMP products (5 µl) were also monitored by 1.5% agarose gel electrophoresis to corroborate the amplification assay.

The efficacy of the LAMP assay designed to amplify *T. muris* DNA in real samples was tested with both pooled faeces and urine samples collected from mice throughout the 80-day infection. Positive (gDNA from *T. muris*) and negative (DNA mix from non-infected mice stool or urine samples) controls were included in all LAMP trials.

## Results

### Monitoring of *T. muris* infection by counting eggs in mice stool samples

**Fig. 1:**
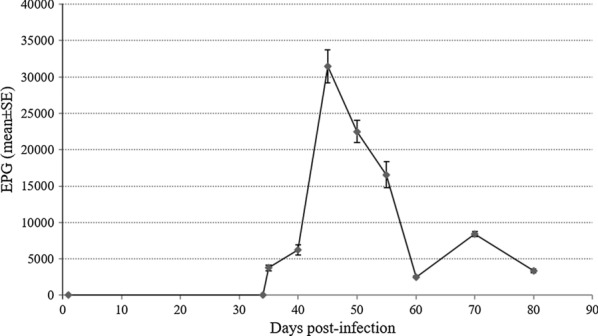
Monitoring of *Trichuris muris* infection by counting eggs in mice stool samples. Mice were experimentally infected with a dose of 300 eggs of the parasite. X axis represent days post-infection and Y axis represent number of eggs per gram of faeces (EPG) and (mean ± SE)

*Trichuris muris* eggs were detected for the first time in faeces (3749 ± 365 EPG) on day 35 p.i. (Fig. 1). Subsequently, the fecal egg count was: 6261 ± 710 EPG on day 40 p.i.; 31,495 ± 2275 EPG on day 45 p.i.; 22,473 ± 1529 on day 50 p.i.; 16,548 ± 1791 EPG on day 55 p.i.; 2494 ± 145 EPG on day 60 p.i.; 8422 ± 363 EPG on day 70 p.i., and 3348 ± 292 EPG on day 80 p.i.

### PCR F3-B3: analytical specificity and limit of detection

**Fig. 2:**
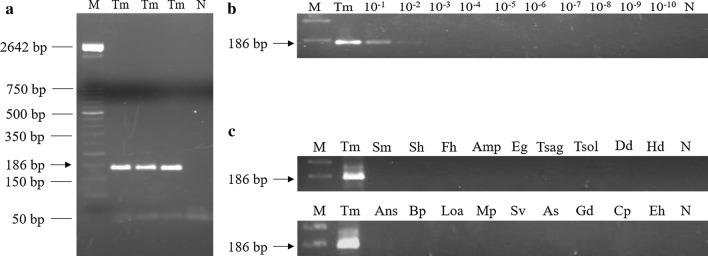
PCR verification, detection limit and specificity using outer primers F3 and B3 for genomic DNA amplification of *Trichuris muris.* **a** Detection limit of the expected 186 bp target length amplicon using PCR F3-B3. Lane M: molecular weight marker (100 bp Plus Blue DNA Ladder); Lane Tm: DNA of *T. muris* (20 ng); Lane N; negative control (ultrapure water, no DNA template). **b** Detection limit of PCR F3-B3. Lane M: molecular weight marker (100 bp Plus Blue DNA Ladder); Lane Tm: DNA of *T. muris* (20 ng); Lanes 10^−1^–10^−10^: 10-fold serial dilutions of *T. muris* DNA. **c** Specificity of PCR F3-B3. Lane M: 50 bp DNA ladder (Molecular weight marker XIII Roche); Lane Tm: DNA of *T. muris* (20 ng); Lanes Sm, Sh, Fh, Amp, Eg, Tsag, Tsol, Dd, Hd, Ans, Bp, Loa, Mp, Sv, As, Gd, Cp and Eh: DNA samples of *Schistosoma mansoni*, *S. haematobium*, *Fasciola hepatica*, *Amphimerus* sp., *Echinococcus granulosus*, *Taenia saginata*, *T. solium*, *Dicrocoelium dendriticum*, *Hymenolepis diminuta*, *Anisakis simplex*, *Brugia pahangi*, *Loa loa*, *Mansonella perstans*, *Strongyloides venezuelensis*, *Ascaris suum*, *Giardia duodenalis*, *Cryptosporidium parvum* and *Entamoeba histolytica,* respectively; Lane N: negative controls (ultrapure water, no DNA template)

The *in silico* expected 186 bp PCR product using outer primers F3 and B3 was successfully amplified from *T. muris* DNA (Fig. 2a). The detection limit obtained by the PCR F3-B3 amplification resulted in 0.2 ng (Fig. 2b). No amplicons of DNA from other parasites included in the analytical specificity trial were obtained (Fig. 2c).

### Setting up the LAMP assay: Whip-LAMP

**Fig. 3:**
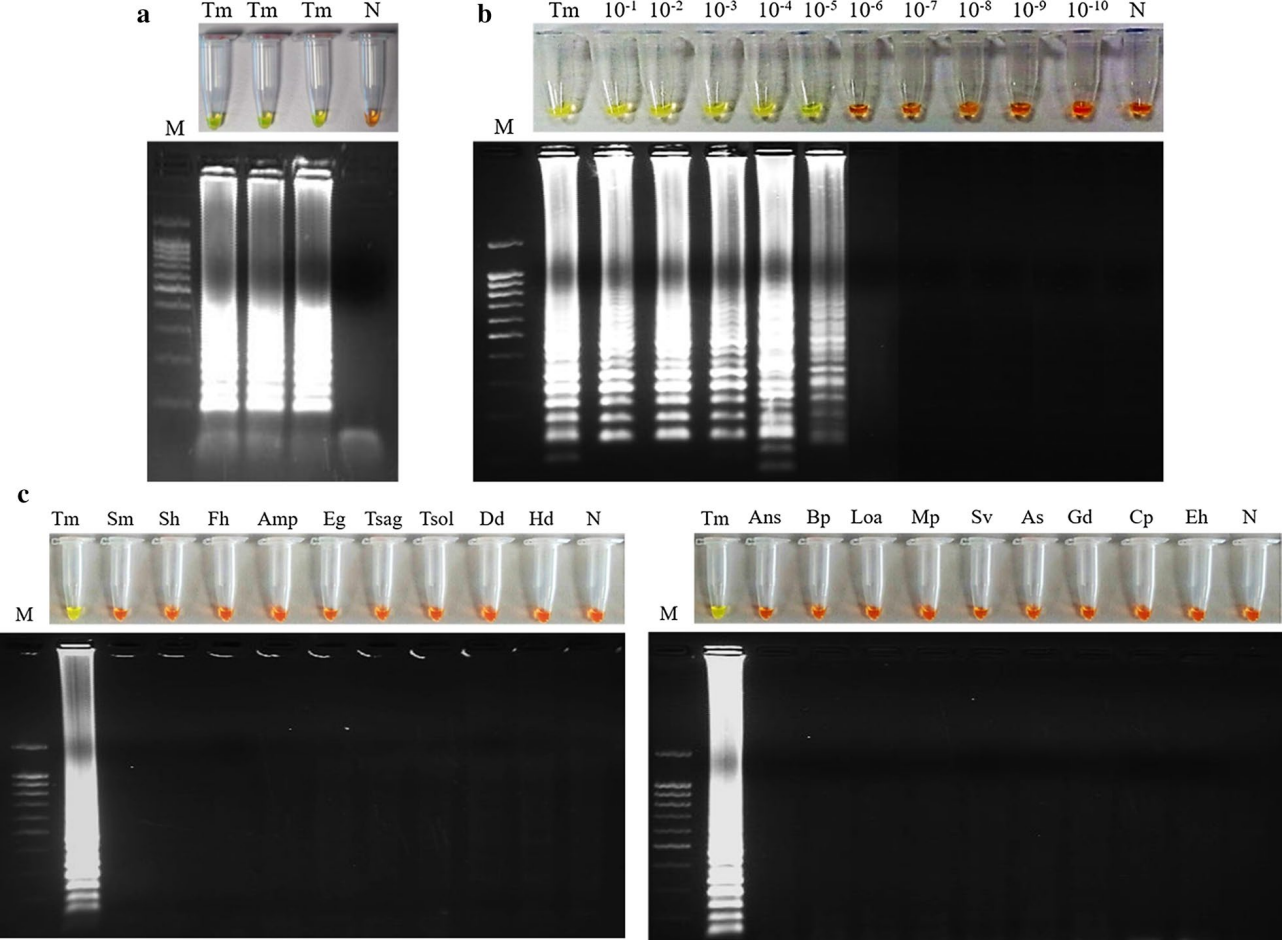
Establishing the Whip-LAMP assay. **a** LAMP results obtained using the set-up Whip-LAMP visualized with the addition of SYBR Green I (up) or agarose gel (down). Lane M: molecular weight marker (100 bp Plus Blue DNA Ladder); Lane Tm: DNA of *T. muris* (20 ng); Lane N: negative control (ultrapure water instead DNA template). **b** Detection limit of the Whip-LAMP assay using serial dilutions of *T. muris* genomic DNA. Lane M: molecular weight marker (100 bp Plus Blue DNA Ladder); Lane Tm: DNA of *T. muris* (20 ng); Lanes 10^−1^–10^−10^: 10-fold serial dilutions of *T. muris* DNA; Lane N: negative control (ultrapure water instead DNA template). **c** Specificity of the Whip-LAMP assay. Lane M: molecular weight marker (100 bp Plus Blue DNA Ladder); Lane Tm: DNA of *T. muris*; Lanes Sm, Sh, Fh, Amp, Eg, Tsag, Tsol, Dd, Hd, Ans, Bp, Loa, Mp, Sv, As, Gd, Cp and Eh: DNA samples of *Schistosoma mansoni*, *S. haematobium*, *Fasciola hepatica*, *Amphimerus* sp., *Echinococcus granulosus*, *Taenia saginata*, *T. solium*, *Dicrocoelium dendriticum*, *Hymenolepis diminuta*, *Anisakis simplex*, *Brugia pahangi*, *Loa loa*, *Mansonella perstans*, *Strongyloides venezuelensis*, *Ascaris suum*, *Giardia duodenalis*, *Cryptosporidium parvum* and *Entamoeba histolytica*, respectively; Lane N: negative controls (ultrapure water instead of DNA template)

After testing different reaction mixtures, temperature conditions and reaction times, the best amplification results (based on turbidity, colour change, intensity of bands in agarose electrophoresis and reproducibility of tests) were obtained when using 1 M betaine combined with supplementary 6 mM MgSO_4_ and incubated at 63 °C for 60 min (Fig. 3a). The reaction mixture, in addition to the specific four primers designed, was set up as the most appropriate for amplification of *T. muris* DNA and was named “Whip-LAMP”. The Whip-LAMP assay was used in all subsequent reactions.

Using the Whip-LAMP, the limit of detection in *T. muris* genomic DNA amplification resulted in 0.0002 ng (2 pg) (Fig. 3b) which was up to 10^3^ times more than the previously obtained by PCR F3-B3. Regarding the specificity, only *T. muris* DNA was amplified; DNA samples from other parasites were never amplified, demonstrating no cross-amplification (Fig. 3c).

### Examination of mice stool and urine samples by PCR F3-B3 and Whip-LAMP

**Fig. 4:**
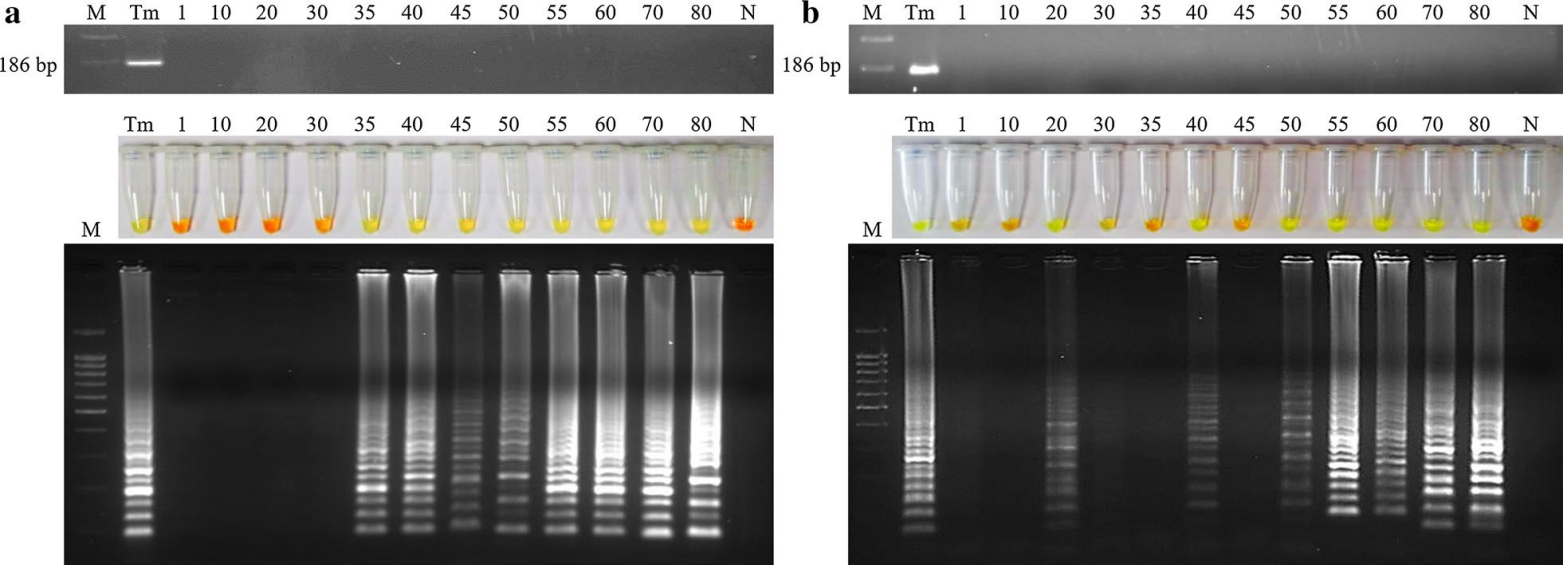
Examination of mice stool and urine samples by PCR (F3-B3) and Whip-LAMP assays. **a** Analysis of pooled stool samples by PCR (F3-B3) (up) and by Whip-LAMP (down). Lane M: molecular weight marker (100 bp Plus Blue DNA Ladder); Lane Tm: positive control (*T. muris* genomic DNA); Lanes N: negative control (pooled DNA samples from faeces from uninfected mice). **b** Analysis of pooled urine samples by PCR (F3-B3) (up) and by Whip-LAMP (down). Lane M: molecular weight marker (100 bp Plus Blue DNA Ladder); Lane Tm: positive control (*T. muris* genomic DNA); Lanes N: negative control (pooled DNA samples from urine from uninfected mice)

Stool samples obtained from the infected mice on days 1, 10, 20, 30, 40, 45, 50, 55, 60, 70 and 80 p.i. were tested by both PCR (F3-B3) and Whip-LAMP. No positive results were obtained by PCR (F3-B3). When testing stool samples by Whip-LAMP, we obtained positive results on day 35 p.i. (when *T. muris* eggs were detected in faeces for the first time) and also in the remaining samples obtained until the end of the experiment at day 80 p.i. (Fig. 4a). Negative controls (pooled DNA samples from faeces from uninfected mice) never amplified as observed by colour and electrophoresis.

PCR (F3-B3) and Whip-LAMP were also performed in urine samples from infected mice on days 1, 10, 20, 30, 40, 45, 50, 55, 60, 70 and 80 p.i. No positive results were obtained by PCR (F3-B3). However, analyses by Whip-LAMP showed amplification results on days 20 (15 days before than onset of *T. muris* eggs in faeces), 40, 50, 55, 60, 70 and 80 p.i. (Fig. 4b). Negative controls (pooled DNA samples from urine from uninfected mice) never amplified. By contrast, we obtained negative results from Whip-LAMP on days 35 p.i. (when eggs were detected for the first time in faeces) and 45 p.i. (when the maximal fecal egg count was observed).

## Discussion

Over the past several years, efforts have been made to use molecular diagnostic methods, particularly qPCR and multiplex-qPCR, to detect soil-transmitted helminths [20, 46–48]. Traditionally, most of these techniques have been restricted to the research setting since they are not easily accessible for low-income countries that are the endemic areas of STH infections. In addition, it has been argued that the development of new diagnostic techniques has been slowed down by the strong focus on drug coverage rather than parasitological monitoring in most soil-transmitted helminthiasis control programmes [49]. Among the species that cause STH infections, *T. trichiura* is the least well studied in the context of molecular-based diagnosis. Bearing in mind that *T. trichiura* causes one of the four major STH infections of man worldwide, with the highest prevalence in children [3], there is a need to carry out research aiming to develop new molecular tools enabling increased precision of diagnostics of trichuriasis. The LAMP method has significant advantages that could be an important alternative of PCR-based methods especially in low-income areas [50]. Furthermore, molecular PCR-based approaches for *Trichuris* spp. DNA detection have been mainly evaluated for stool samples, and until now, no urine samples have been used for molecular diagnostic purposes. As for other gut-dwelling helminths, such as *Ascaris lumbricoides* and cestodes, it would be of interest to detect transrenal cell-free DNA (cfDNA) of *Trichuris* spp. [6], since urine samples have a number of advantages over stool samples, including better handling, management and storage. In this study, we used an experimental *T. muris* mouse model to collect well-defined stool and, for the first time, urine samples to test a new LAMP assay for diagnosing trichuriasis. The mouse whipworm, *T. muris*, has traditionally served as a useful model of the human parasite *T. trichiura* and more recently, the description of both parasite genomes supports the use of *T. muris* as a suitable model of human trichuriasis [51].

In our study, a direct copro-parasitological test by counting EPG was used for monitoring infection and to compare results with molecular methods. We detected eggs in faeces for the first time on day 35 p.i., thus indicating a similar dynamics of *T. muris* infection to that previously reported by this parasite in other models of experimental mouse infection [52–54].

To design primers for LAMP, a number of available sequences of the *18S* rRNA gene for both *Trichuris muris* and *T. trichiura* were retrieved from databases. This gene is highly conserved among the different *Trichuris* species and has already been used successfully in previous studies on *T. trichiura* DNA detection [55]. A multiple alignment with the selected sequences generated a 1910 bp consensus sequence which showed 93% identity with the sequence of the *18S* rRNA gene of *T. muris* and 94% with the partial sequence of the *18S* rRNA gene of *T. trichiura*, so it was selected *in silico* as a good candidate to design specific primers for the simultaneous detection of both parasite species.

Initially, we established the proper operation, sensitivity and specificity of both PCR (using the external primers F3-B3) and LAMP (using the set of four primers: Whip-LAMP) in the amplification of the selected *Trichuris* spp. DNA target sequences. Both molecular assays were highly specific for *Trichuris* spp. since no cross-amplification was detected when DNA samples from other parasites were tested, including a number of helminths and protozoans that could hypothetically appear in human stool samples. Regarding sensitivity, the limit of detection using Whip-LAMP (0.0002 ng) is up to 10^3^ times lower than the obtained by PCR F3-B3 (0.2 ng). These results are in line with the described higher sensitivity usually obtained with LAMP compared to a conventional PCR reaction [56].

When the PCR F3-B3 was tested using both stool and urine samples collected from infected mice, no amplicons were obtained but the predicted 186 bp PCR product was always successfully amplified from *T. muris* genomic DNA (gDNA) used as a positive control. In this regard, it seems logical to consider that the absence of positive results in samples cannot be due to lack of operation in the PCR. In previous research by our group, we obtained similar results regarding the poor performance of PCR F3-B3 compared to the LAMP assay when using biological samples from both an experimental rodent model for the detection of *Strongyloides venezuelensis* [34] and human samples for the detection of *Amphimerus* sp. [57]. The decrease in the sensitivity of the PCR in the analysis of biological samples could be due to an inhibition of *Taq* polymerase by different substances commonly found in faeces and urine probably not entirely removed with DNA extraction methods prior to PCR [58], and which are not present in gDNA from the parasite. Nonetheless, in this study the PCR is not emphasized, because of its very low performance and the handicap of application in poorly equipped laboratories in endemic areas of developing countries.

However, using the Whip-LAMP assay we obtained positive results both in stool and urine samples. The performance, inhibitor tolerance, sensitivity and robustness of the LAMP assay for diagnostic applications in comparison with conventional PCR has already been reported [59, 60]. When testing stool samples, we obtained Whip-LAMP positive results continuously from day 35 p.i. until the end of infection at day 80 p.i., correlating with microscopy-based monitoring of infection by counting the number of EPG.

Analysis of urine samples from infected mice showed Whip-LAMP positive results on day 20 p.i. (thus, 15 days before the onset of *T. muris* eggs in faeces) and on days 40, 50, 55, 60, 70 and 80 p.i. A similar occurrence was observed in our study on the detection of *S. venezuelensis* DNA in urine samples from experimentally infected rats [34]. In that study, parasite cfDNA was also detected in urine samples a few days before the onset of eggs in faeces, probably as a consequence of destroying larvae passing through the lungs and ending up in urine. However, unlike *Strongyloides* spp., *Trichuris* species do not migrate through the lungs in their life-cycle [6]. The presence of *T. muris* cfDNA in urine a few days before the appearance of eggs in faeces could be due to the four classical larval moults of nematodes (L1, L2, L3 and L4) in the epithelial layer to reach adulthood in the caecum and proximal colon that occur over a period of 9–32 days p.i.: first moult (L1-L2 larvae occur on days 9–11 p.i.), second moult (L2-L3 larvae occur on day 21 p.i.), third moult (L3-L4 larvae occur on days 24–28), and fourth moult (L4 larvae and adults occur on days 29–32 p.i.) [12]. Additionally, moults may occur at slightly different time points depending on the host strain [49].

Unexpectedly, we did not obtain Whip-LAMP positive results in urine samples from days 35 p.i. (when eggs were microscopically detected for the first time in faeces) and 45 p.i. (when the maximal EPG was detected). Intriguingly, we also observed something similar in the above-mentioned study with *S. venezuelensis*, obtaining a negative result in urine on the same day when the maximal faecal egg count was reached [34]. As we also pointed out at the time, we have no information available to explain this fact, and at this stage, we can only speculate on the possibility of some events related to the dynamics of the life-cycle of the parasite.

## Conclusions

To the best of our knowledge, we report for the first time the development of a novel LAMP assay (Whip-LAMP) for sensitive detection of *T. muris* DNA in both stool and urine samples in a well-established mouse experimental infection model. The successful amplification of *T. muris* cfDNA in urine samples by Whip-LAMP, the high similarity between *T. muris* and *T. trichiura* and the advantages of handling the urine samples in comparison to patients’ stool samples, should make us consider the possibility of starting to use clinical urine specimens for both molecular diagnosis and field-based studies of human trichuriasis, when possible. Further studies with clinical urine samples are needed.

## Data Availability

All data generated or analysed during this study are included in this published article.

## Abbreviations

qPCR: quantitative polymerase chain reaction
18S rRNA: *18S* ribosomal RNA
LAMP: loop-mediated isothermal amplification
WHO: World Health Organization
STHs: soil-transmitted helminths
DALYs: disability-adjusted life-years
ITS: internal transcribed spacer
gDNA: genomic DNA
BLAST: Basic Local Alignment Search Tool
